# Estimation of the Surface Dose in Breast Irradiation by the Beam Incident Angle and the 1 cm Depth Dose

**DOI:** 10.3390/jcm11082154

**Published:** 2022-04-12

**Authors:** Tsung-Yu Yen, Kai-Cheng Chuang, Hsiao-Mei Fu, Chen-Ju Feng, Ke-Yu Lien, Shih-Ming Hsu

**Affiliations:** 1 Department of Biomedical Imaging and Radiological Sciences, National Yang Ming Chiao Tung University, Taipei 112304, Taiwan; yms851115@yahoo.com.tw (T.-Y.Y.); k1a2i3oscar@gmail.com (K.-C.C.); iris450399@hotmail.com (C.-J.F.); cacacaca40910@gmail.com (K.-Y.L.); 2 Medical Physics and Radiation Measurements Laboratory, National Yang Ming Chiao Tung University, Taipei 112304, Taiwan; 3 Department of Radiation Oncology, MacKay Memorial Hospital, Taipei 251020, Taiwan; moos1028@yahoo.com.tw; 4 Hospice and Palliative Care Center, MacKay Memorial Hospital, Taipei 251020, Taiwan; 5 Medical Physics Graduate Program, Louisiana State University, Baton Rouge, LA 70803, USA

**Keywords:** breast radiotherapy, surface dose, anthropomorphic phantom

## Abstract

To develop a method of estimating surface dose in whole breast irradiation, we used an anthropomorphic phantom with accessories for the simulation of different breast sizes. The surface points, which are measured by TLDs, are set along with two main directions, superior-inferior and medial-lateral. The incident angle between the photon beam and the surface and the doses at 1 cm beneath the surface at every point are assessed by a computerized treatment planning system (cTPS). With the prescription dose of 200 cGy, the average surface doses under tangential irradiation are 97.73 (±14.96) cGy, 99.90 (±10.73) cGy, and 105.26 (±9.21) cGy for large, medium, and small breast volumes, respectively. The surface dose increased in the model of small breast volume without significance (*p* = 0.39). The linear analysis between surface dose and the incident angle is y = 0.5258x + 69.648, R^2^ = 0.7131 (x: incident angle and y: surface dose). We develop the percentage of skin surface dose with reference to a depth of 1 cm (PSDR1cm) to normalize the inhomogeneous dose. The relationship between incident angle and PSDR1cm is y = 0.1894x + 36.021, R^2^ = 0.6536 (x: incident angle and y: PSDR1cm) by linear analysis. In conclusion, the surface dose in whole breast irradiation could be estimated from this linear relationship between PSDR1cm and incident angle in daily clinical practice by cTPS. Further in vivo data should be studied to verify this formula.

## 1. Introduction

Breast cancer is the most common female malignancy disease in Taiwan, with approximately 15,000 new cases diagnosed and over 2000 annual deaths in 2019 [1]. Most early-stage breast cancer patients choose to receive breast conservation treatment, including lumpectomy followed by adjuvant radiation therapy (RT) [2]. Patients typically receive 4–6 weeks of radiotherapy to the entire breast with or without regional lymph nodes. However, breast RT could lead to adverse skin effects such as radiation dermatitis, which is the most common acute side effect [3,4]. Radiodermatitis, which includes dry desquamation, erythema, and moist desquamation, is a significant issue in clinical care. When encountering these side effects, patients and oncologists experience a lot of trouble during treatment, including delay or interruption of RT, diminished aesthetic appeal, and reduced quality of life [5].

It is also known that greater surface doses give rise to more skin adverse effects [6]. Therefore, radiation oncologists and medical physicists should adequately balance between target and skin doses. In addition, a computerized treatment planning system (cTPS) is commonly used for dose calculation and optimization. Nevertheless, the cTPS used in routine clinical practice does not reflect the exact dose to the surface skin region. Therefore, based on the official report of Task Group No. 53 from the American Association of Physicists in Medicine (AAPM) [7], there is up to a 20% difference between the measurement of the ion chamber and TPS calculation in the dose in the build-up region and the surface.

At this surface depth, the gradient of the dose distribution is so high that it is difficult to measure and evaluate the skin dose. It was reported that the relative dose increases from 14% to 43% within the first millimeter in a 6 MV photon beam with a field size of 10 × 10 cm^2^ [8]. Previous studies have shown that thermoluminescent dosimeters (TLD), Gafchromic film, and metal oxide semiconductor field effect transistors (MOSFET) could be proper dosimeters for measuring the surface dose [6,9,10].

Some articles report that the surface dose is related to the incident angle under the setting of a single direction for a rectangular cuboid phantom [11]. Theoretically, a larger incident angle results in a higher surface dose than a smaller angle. In other words, a vertical beam irradiated to a rectangular cuboid phantom could give the smallest surface dose compared to any other oblique angle [11]. However, clinical whole breast irradiation is not similar to this simple situation. Based on the dome-shaped breast under the traditional two opposite tangential beams, every point on the skin has a different incident angle between the surface and irradiation beams. Moreover, various breast sizes also lead to different incident angle distributions over the whole breast surface.

The purpose of this study is to investigate the impact of breast volume and different incident angles on the surface dose by using an anthropomorphic phantom irradiated under the conventional external beam technique. The exact skin surface dose is measured by TLDs. We analyze the surface dose and determine the relationship between the incident angle and surface dose. Based on this relationship, we try to develop a predicted formula for breast surface dose which could be easily applied in the clinic.

## 2. Materials and Methods

### 2.1. Linear Accelerator (LA), Anthropomorphic Phantom, and cTPS

In this study, all irradiation experiments were performed by a 6 MV photon beam produced from a LA as Varian Clinac iX (manufactured by Varian Medical Systems, Palo Alto, CA, USA). An Alderson Rando anthropomorphic phantom (manufactured by Alderson Research Laboratories, Inc., Long Island City, NY, USA) with three layers of breast accessories was applied to simulate whole-breast irradiation for different breast sizes.

Three situations were set based on the anthropomorphic phantom breast accessories for simulating different breast volumes. First, by directly putting it on the chest wall, a large-size breast was simulated using the whole three layers of breast accessories. Next, a medium-size breast was set using the upper two layers of breast accessories, and a single top layer was applied to mimic the smallest breast volume. Finally, all three breast size situations were set on the left side of the anthropomorphic phantom chest wall.

Computerized tomography (CT) simulation scans (Philips Brilliance Big Bore CT simulator, manufactured by Philips, Amsterdam, Netherlands) for the radiotherapy plan were applied. Based on the CT scan simulation image, radiation oncologists drew the clinical target volume (CTV), planning target volume (PTV), and neighboring normal organs. The CTVs of these three breast sizes were 531.3, 409.4, and 104.3 mL. The prescription dose was 200 cGy. Medical physicists optimized the radiotherapy plan by the 3-dimensional technique as opposite two tangential beams under the cTPS (Eclipse planning system, Version 13.0; manufactured by Varian Medical Systems, Palo Alto, CA, USA). Medical physicists can choose wedges that are modified from a multi-leaf collimator system by cTPS to better optimize the RT plan. After the medical physicists completely planned the radiotherapy treatment, the physician must check and confirm the plan before administering irradiation.

### 2.2. Thermoluminescent Dosimeter

**Figure 1:**
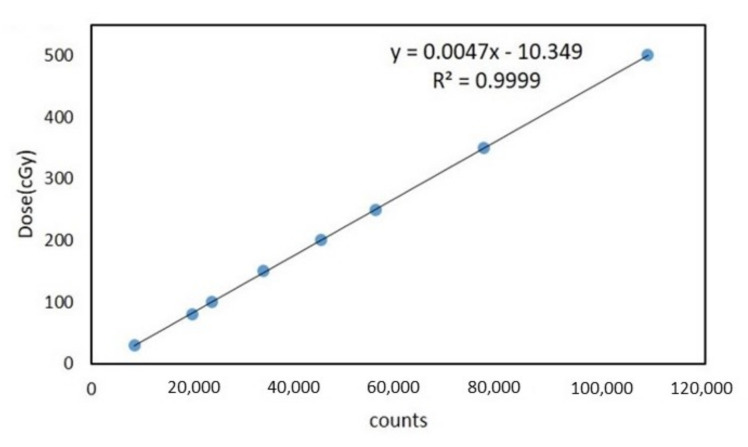
A TLD calibration curve is generated by irradiating a dose range of 30–500 cGy.

Ultrathin thermoluminescent dosimeter (TLD) chips (GR200F, surface area 5 × 5 mm^2^, nominal thickness 5 mg/cm^2^; manufactured by Solid Dosimetric Detector & Method Laboratory, SANGE Technologies Inc., Beijing, China) were applied for the measurement of surface dose on the designated measuring points over the anthropomorphic breast accessories. The ultrathin TLD chips have high sensitivity and a tissue-equivalent atomic number (Z = 8.2). Before using the TLDs to assess the skin dose, the calibration curve of the TLDs was generated by irradiating a dose range of 30–500 cGy to the first eight TLDs. The calibration curve is shown in Figure 1. Then the TLDs were placed at the designed measuring points on the breast accessories. After irradiation, the TLDs were analyzed by a Rexon UL-320 TLD Reader (manufactured by Rexon UL-32, Beachwood, OH, USA), and the readout program followed the suggested instructions of the manufacturer. The results were recorded and adjusted by the previous calibration curve. After measurement, the TLDs were annealed at 240 °C for 10 minutes. All TLD-measured points on the breast accessories’ surface were performed three times.

### 2.3. Surface Dose Measurement in Different Beam Angles by Rectangular Cuboid Phantom

**Figure 2:**
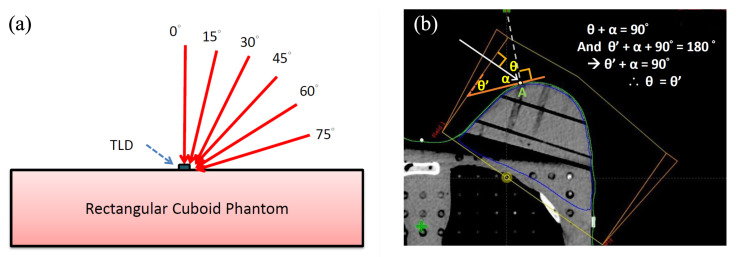
(**a**) Illustration of a one-direction beam from the different incident angles. TLD was put on the surface of the cuboid phantom. (**b**) The theoretical incident angle between the beam angle (white arrow line) and point A is θ (orange line: the tangent line at point A). Under the derivation described above, the angle θ’ is equal to angle θ. It is more easily checked by cTPS for angle θ’, which is just between the tangent line and the field plane line.

A rectangular cuboid solid-water phantom was set for the measurement incident angle—the surface effect. Surface doses of this acrylic phantom were measured at 0°, 15°, 30°, 45°, 60°, and 75° incident angles at a 100-cm source-to-axis distance (SAD) (Figure 2a). The prescribed dose was 200 cGy at a 1.5 cm depth.

### 2.4. Measuring Points of Surface Dose Measurement for Different Breast Sizes Using Phantom Accessories

**Figure 3:**
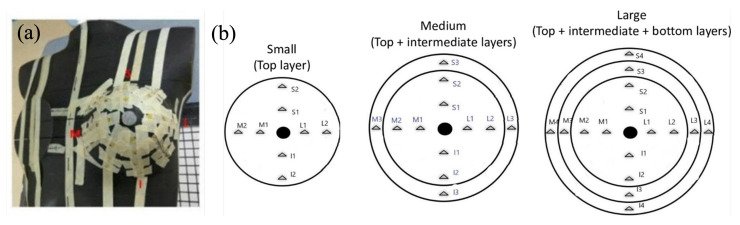
(**a**) The front view of the anthropomorphic phantom with TLDs. (**b**) The illustration of measured points on three different breast accessories.

Measuring points on the surface of different breast sizes were set on two cross axes (Figure 3). The designated points were mapped as medial-lateral and superior-inferior axes based on the two traditional directions. The cross point of these two directions was the location of the nipple point in the center of the breast accessories. The total number of measuring points over large, medium, and small breast accessories was 16, 12, and 8 points, respectively. However, the point of the nipple was not measured as its location was concave and may cause measuring bias.

### 2.5. Measurement of Incident Angle and Irradiation Dose at Each Designated Point by Computerized TPS

The designated measuring points are marked on the CT simulation scan for the realization of the CT image. The point dose at 1 cm under the skin surface for every designated measuring point calculated by cTPS was recorded. The incident angle is defined as the irradiation beam. The rectangle angle from the surface to every measuring point is also drawn and calculated by cTPS. After the mathematical rules of triangles and rectangle angles, we found a simple method of estimating the incident angle (Figure 2b). For each measurement using TLDs, the same procedures were repeated three times.

### 2.6. Percentage of Skin Surface Dose Reference to a Depth of 1 cm (PSDR1cm)

The percentage of skin surface dose was calculated using the following formula:

$$\mathrm{PSDR}1\mathrm{cm}=\frac{\left(\mathrm{Measured}\;\mathrm{skin}\;\mathrm{surface}\;\mathrm{dose}\;\mathrm{by}\;\mathrm{TLD}\right)}{\left(\mathrm{Reference}\;\mathrm{dose}\;(1\mathrm{cm}\;\mathrm{depth}\;\mathrm{under}\;\mathrm{skin})\;\mathrm{by}\;\mathrm{TPS}\right)}\;\times 100\%$$

### 2.7. Statistical Methods

Results are presented as mean or mean ± standard deviation (SD). Comparisons between different breast volumes were made using one-way ANOVA. Linear regression analysis was applied to assess the relationship between incident angle vs. surface dose and incident angle vs. PSDR1cm. A p-value of less than 0.05 was considered statistically significant.

## 3. Results

### 3.1. Surface Dose on a Rectangular Cuboid Solid-Water Phantom and Its Relationship to the Incident Angle

**Figure 4:**
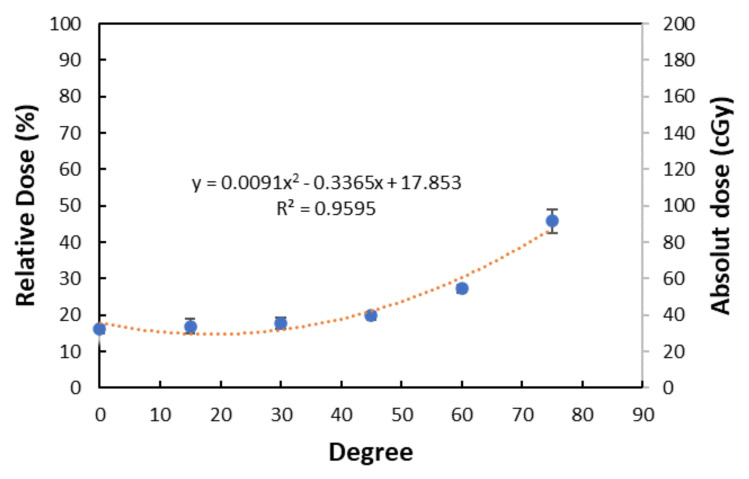
The surface dose by different incident angles. The trend line is a binomial regression, as described above.

A rectangular cuboid solid-water phantom was applied to understand the relationship between the incident angle and surface dose. The surface doses are measured with the TLD placed on the surface of the homogeneous solid-water rectangular cuboid phantom for various incidental angles (Table 1). The TLD dose increased from 32.27 cGy to 91.51 cGy as the incidental angle increased from 0° to 75°. The results indicated that the surface dose increased when the incident angle became more oblique (Figure 4). The trend line is y = 0.00091x^2^ – 0.3365x + 17.853 (R^2^ = 0.9594) when y represents the relative dose and x represents the incident angle.

### 3.2. Surface Dose to Breast Accessories Measured by TLDs

**Figure 5:**
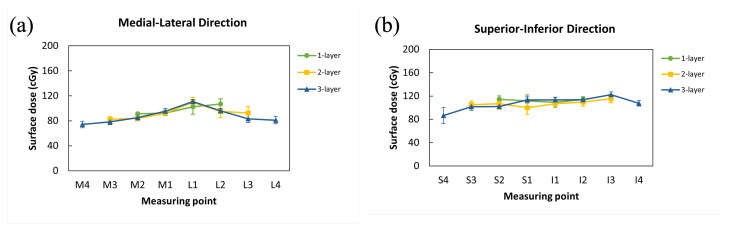
The dose distribution among three different breast accessories. (**a**) Medial-lateral direction and (**b**) superior-inferior direction.

The results of surface doses measured by TLDs at each measuring point for three different breast volumes are shown in Table 2 and Table 3. With a prescribed dose of 200 cGy, the skin surface dose measured by TLDs ranged between 74.12 and 122.18 cGy. The averaged surface doses measured by TLDs were 97.73 ± 14.96 cGy on the 3-layer accessories, 99.90 ± 10.73 cGy on the 2-layer accessories, and 105.26 ± 9.21 cGy on the smallest 1-layer accessories. Although not statistically significant, the average surface dose measured by TLDs was slightly higher on the smallest breast than on the other two (*p* = 0.394) (Table 2 and Table 3 and Figure 5).

### 3.3. Relationship between Incident Angle and Surface Dose among Various Sizes of Breast Accessories

**Figure 6:**
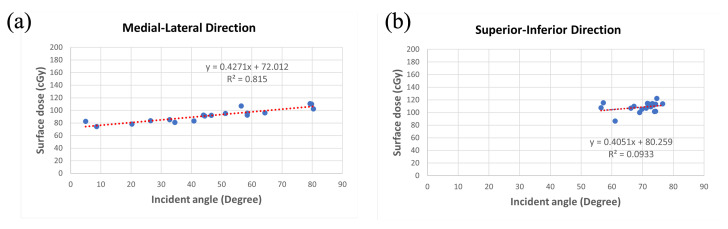
The relationship between surface dose and incident angle. Both the trendlines (red dashed lines) are under linear regression as above. (**a**) The medial-lateral direction and (**b**) the superior-inferior direction.

The results of skin incident angle and surface dose at each measuring point are shown in Table 2 and Table 3. The relationship between incident angle and surface dose for two-direction is depicted in Figure 6. Under linear analysis, we found a result of y = 0.4051x + 80.259, R^2^ = 0.0933 for the superior-inferior direction and y = 0.4271x + 72.012, R^2^ = 0.815 for the medial-lateral direction when x represents the incident angle (degree) and y represents surface dose (cGy).

### 3.4. Relationship between Incident Angle and PSDR1cm among Various Sizes of Breast Accessories

**Figure 7:**
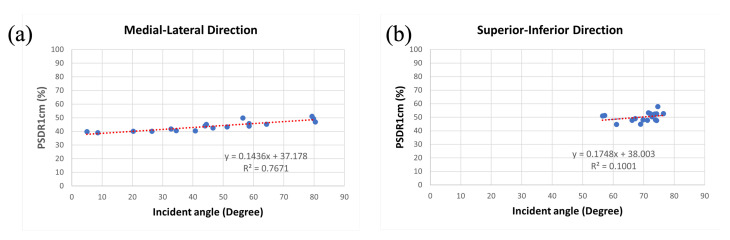
The relationship between PSDR1cm and incident angle. Both the trendlines (red dashed lines) are under linear regression. (**a**) The medial-lateral direction and (**b**) the superior-inferior direction.

We developed a formula, PSDR1cm, as above, for the normalization effect of inhomogeneous inside breast volume under tangential irradiation. The results are also presented in Table 2 and Table 3. Under linear analysis, we found a results of y = 0.1748x + 38.003, R^2^ = 0.1001 for the superior-inferior direction and y = 0.1436x + 37.178, R^2^ = 0.7671 for the medial-lateral direction when x represents PSDR1cm (%) and y represents the surface dose (cGy) (Figure 7).

## 4. Discussion

The breast surface doses under opposite tangential irradiation beams have been studied in previous literature, which showed that the dose ranges from 40% to 65% of the prescribed dose [10,12,13,14]. The reason that the results obtained in different studies varied substantially may be attributed to factors such as the phantom material, the shape of the breast, the dosimetry tools for measurement, and the beam energy. However, most of the aforementioned studies were performed using a self-designed phantom or measured in vivo. The disadvantage of a self-designed cylinder phantom is that the phantom is too symmetric to simulate humans’ various shapes of the breast in the real world [10]. The in vivo study of breast surface dose is the best way to reflect the actual surface dose compared to phantom studies. However, it is not easy to attach numerous measuring points to dosimeters at the same point every day. This rigid anthropomorphic phantom we applied could be an alternative way for quickly setting up for every irradiation and performing them with numerous measuring points on its surface.

Therefore, we used a standard anthropomorphic phantom with official breast accessories to mimic natural human beings instead of a cylinder phantom. The breast accessories are also made by simulating the human body, and they are asymmetrically dome-shaped, which is not like a cylindrical, symmetric phantom. Moreover, data on an anthropomorphic phantom with various sizes of breast accessories are lacking in previous literature.

Previous studies revealed that female breast volume could be related to multiple factors, such as body mass index, age, etc. [15,16]. Furthermore, some previous studies revealed that breast size is an independent factor affecting acute skin toxicity. Patients with a large breast volume are more likely to suffer from more significant acute and chronic toxicities [17,18,19,20]. Based on our anthropomorphic study, small breast sizes receive a slightly greater surface dose than bigger breast volumes, and our findings are not similar to previous studies. Several reasons could be hypothesized, as follows. First, a larger breast bust could result in more skin folds when the patient is supine in daily treatment. The skin folds can enhance the dose deposition on the skin surface. This fold deformity phenomenon does not happen in our rigid anthropomorphic phantom. Second, the irradiation field of clinical whole breast radiotherapy is not only for the whole breast but also for the regions of related axillary lymph node areas. The surface dose of related axillary irradiation is not evaluated in our anthropomorphic phantom study. Finally, the radiotherapy in some previous studies was delivered by Cobalt-60 or planned by a 2-D radiotherapy technique without a CT simulator or TPS [17,18], which was different from our study and could be a confounding factor for surface dose.

Much literature mentions that the incident angle between the single irradiation beam and material surface could significantly affect the surface dose. Dr. Qian and his colleagues showed that the incident angle does affect the surface dose. That is to say, the surface dose increases while the incident angle increases [21]. Consistent with their findings, the data obtained in our study also showed similar results when using a rectangular cuboid solid-water phantom. However, some articles also point out that the opposite two beams might decrease this effect in the cylinder phantom. By using our anthropomorphic phantom and delivering opposite tangential two-beam irradiation, the relationship between the incident angle and surface dose is analyzed. Our result supposes only a slight elevation in surface dose when the incident angle increases. This is also in agreement with the measured data on the chest-simulated Perspex phantom by Nakano et al. [10]. This study placed only five measuring points in the medial-lateral direction on a non-anthropomorphic cylinder phantom. Compared with the research of Nakano et al. [10], our data showed that the incident angle plays a minor role in surface skin dose due to the varying incident angle across various breast sizes.

**Figure 8:**
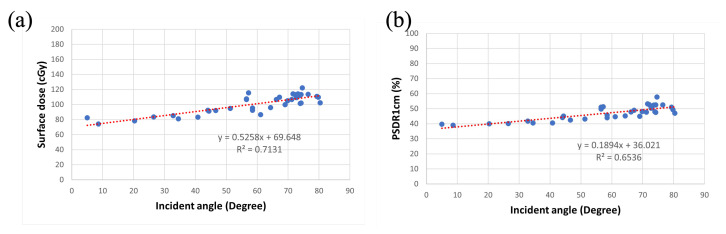
The relationship between surface dose, PSDR1cm and incident angle in both directions. Both the trendlines (red dashed lines) are under linear regression. (**a**) is the surface dose vs. the incident angle and (**b**) is PSDR1cm vs. the incident angle.

Given that breast irradiation is not homogeneous inside the whole breast volume, higher doses are often located around the central region near the nipple area after TPS optimization. This phenomenon could result in a higher surface dose around the central area. Therefore, we developed a unique formula to represent the surface dose as a fraction of the reference point dose, which is named PSDR1cm. It is defined as the percentage fraction of the surface dose divided by a reference dose at 1 cm depth below the designated surface measuring point. We chose this reference location because this point dose was easily checked in cTPS, and it is nearly equal to the real absorbed dose that has been investigated by Moncion and his colleagues [22]. After this correction, the effect of incident angle on the surface dose is diminished. We also found a better linear relation in the medial-lateral direction than in the superior-inferior direction. The values of R^2^ are near to 1 in the former and near to 0 in the latter. This could be a result of the narrow range of incident angles in superior-inferior direction. Therefore, we put all measured data regarding surface dose and PSRD1cm together and analyzed them (Figure 8). The linear analysis results are y = 0.5258x + 69.648, R^2^ = 0.7131 when x represents the incident angle (degree) and y represents the surface dose (cGy) and y = 0.1894x + 36.021, R^2^ = 0.6536 when x represents PSDR1cm (%) and y represents the surface dose (cGy). According to these R^2^ values, both are reliable and valid linear trend line.

Dr. McDermott has also studied the breast surface dose by various sizes of breast volume. He analysed the statistics by simulation of the breast as an eclipse and the polar angle [23]. However, it is difficult to calculate the defined polar angle from SAD and two axes of the simulated eclipse. We developed the predicted equation based on the relationship between PSDR1cm, the angle of incidence, and the dose at 1 cm depth from the surface by cTPS, which are all more easily applied in daily work. PSDR1cm could be represented as the following equation:

PSDR1cm=0.1894×(angleofincidence)+36.021

Therefore, the equation of predicted surface dose is shown as follows:

Predictedsurfacedose=PSDR1cm×(1cmdepthdosefromskinbycTPS)

## 5. Conclusions

Based on our study, the surface dose under tangential breast irradiation can be predicted easily by the angle of incidence and 1 cm depth dose on cTPS in daily practice. Further in vivo studies are planned in the future.

## Figures and Tables

**Table 1:** The results of incident angle and surface dose under one direction to the rectangular cuboid phantom. The average of the absolute surface dose is 47.92 ± 3.39 (cGy).

Incident Angle (Degree)	Absolute Dose of Surface (cGy)	Relative Dose of Surface (%)
0	32.27 ± 2.18	16.14 ± 1.09
15	33.91 ± 4.21	16.96 ± 2.15
30	35.67 ± 3.11	17.84 ± 1.56
45	39.65 ± 2.29	19.83 ± 1.15
60	54.53 ± 2.18	27.27 ± 1.09
75	91.51 ± 6.35	45.76 ± 3.18

**Table 2:** The results of surface dose, incident angle and PSDR1cm in the medial-lateral direction on three different breast accessories.

Layer(s) of Accessory	Measuring Point	Surface Dose (cGy)	Dose at 1 cm Depth (cGy)	PSDR1cm (%)	Incident Angle (Degree)
	M1	92.32 ± 3.97	210.2	43.92	58.5
1-layer	M2	91.23 ± 1.63	202.3	45.10	44.4
(small)	L1	102.26 ± 11.61	217.6	46.99	80.4
	L2	106.84 ± 8.37	214.5	49.81	56.5
	M1	92.15 ± 0.99	217.1	42.45	46.6
	M2	83.63 ± 3.18	208.4	40.13	26.5
2-layer	M3	82.50 ± 3.93	207.6	39.74	5.0
(moderate)	L1	109.76 ± 7.79	222.1	49.42	79.8
	L2	94.90 ± 9.85	219.7	43.20	51.3
	L3	92.27 ± 9.99	209.4	44.07	44.0
	M1	95.38 ± 4.50	208.5	45.75	58.5
	M2	85.14 ± 5.16	203.6	41.82	32.8
	M3	78.26 ± 4.31	195.5	40.03	20.3
3-layer	M4	74.12 ± 5.25	190.3	38.95	8.6
(big)	L1	110.72 ± 3.47	217.0	51.02	79.3
	L2	96.02 ± 3.90	212.2	45.25	64.3
	L3	83.19 ± 5.99	205.6	40.46	40.8
	L4	80.93 ± 5.90	199.6	40.55	34.5

**Table 3:** The results of surface dose, incident angle and PSDR1cm in the superior-inferior direction on three different breast accessories.

Layer(s) of Accessory	Measuring Point	Surface Dose (cGy)	Dose at 1 cm Depth (cGy)	PSDR1cm (%)	Incident Angle (Degree)
	S1	111.71 ± 10.37	212.9	52.4	73.6
1-layer	S2	114.25 ± 6.09	214.7	53.3	71.6
	I1	102.26 ± 11.61	217.4	50.3	72.7
	I2	114.20 ± 4.57	220.1	51.9	73.2
	S1	99.87 ± 11.30	222.5	44.9	69.0
	S2	106.78 ± 9.75	223.6	47.8	66.2
2-layer	S3	105.05 ± 6.34	218.6	48.1	69.8
	I1	106.70 ± 2.98	223.7	47.7	71.2
	I2	109.68 ± 7.37	224.5	48.9	67.2
	I3	115.51 ± 7.03	225.3	51.3	57.2
	S1	113.28 ± 5.37	214.8	52.7	72.1
	S2	102.05 ± 2.57	214.5	47.6	74.2
	S3	101.57 ± 6.23	211.7	48.0	73.9
3-layer	S4	86.44 ± 13.86	193.6	44.6	61.1
	I1	113.24 ± 4.68	215.5	52.5	74.2
	I2	113.66 ± 3.10	216.2	52.6	76.5
	I3	122.18 ± 4.91	211.2	57.8	74.6
	I4	107.56 ± 4.81	211.0	51.0	56.5

## Data Availability

The corresponding author has full control of the primary data and can be reviewed by the journal if requested.
